# The “flex-and-resheath” technique for improving transpapillary biopsy of the distal biliary tract

**DOI:** 10.1055/a-2952-8543

**Published:** 2026-09-22

**Authors:** Kohei Okamoto, Norimitsu Uza, Masahiro Tsujimae, Arata Sakai, Takashi Kobayashi, Atsuhiro Masuda, Yuzo Kodama

**Affiliations:** 1 Division of GastroenterologyDepartment of Internal Medicine 592910 Kobe University Graduate School of MedicineKobeHyogoJapan

Endoscopic retrograde cholangiopancreatography (ERCP)–guided biopsy of biliary lesions can be technically challenging. A tapered-tip sheath system (EndoSheather; Piolax, Yokohama, Japan) combined with standard-sized forceps (Radial Jaw; Boston Scientific Japan, Tokyo, Japan; Fig. 1 ) enables repeated biopsies through a reliable biliary access route. 1​ For distal bile duct lesions, however, the combined forceps–sheath configuration can restrict forceps flexibility by increasing shaft rigidity, making target biopsy technically difficult. 2​ We describe a “flex-and-resheath” technique that restores forceps flexibility while maintaining the established biliary access route, thereby improving tissue sampling from these lesions ( Video 1 ).

**Fig. 1:**
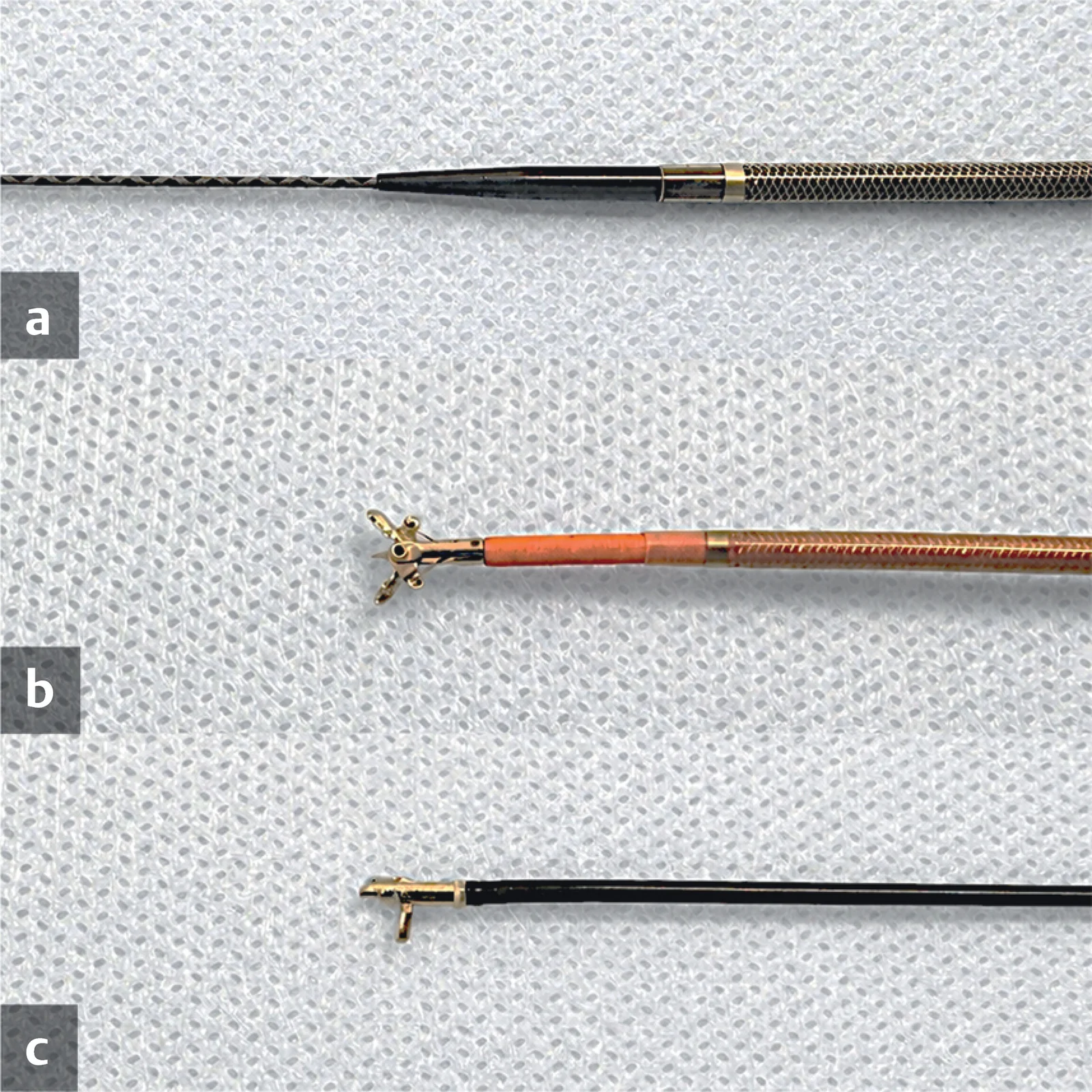
Devices used for ERCP-guided biopsy. ( **a** ) An EndoSheather, a tapered-tip sheath system consisting of an inner catheter and an outer sheath. ( **b** ) Radial jaw, standard-sized forceps with a 5.4-mm opening width, inserted into the outer sheath. ( **c** ) Conventional biliary biopsy forceps with a smaller opening width.

**Video 1** The “flex-and-resheath” technique restores forceps flexibility while preserving biliary access for repeated biopsies of distal bile duct lesions.


A 60-year-old woman was referred for further evaluation of suspected distal biliary malignancy ( Fig. 2 ) after nondiagnostic ERCP-guided biopsy and cytology at another hospital. During repeat ERCP, cholangiography showed a filling defect extending from the ampulla to the distal bile duct ( Fig. 3a ). After EndoSheather insertion, mapping biopsy from the biliary confluence was performed. 1​ For the primary tumor, the outer sheath was temporarily withdrawn into the endoscope while the forceps remained within the bile duct, allowing a curved trajectory toward the lesion ( Fig. 3b ). Subsequent resheathing over the forceps preserved biliary access and enabled repeated biopsies ( Fig. 3c ). Temporary sheath withdrawal restored forceps-shaft flexibility, as illustrated by the procedural sequence and both the sheathed and unsheathed configurations ( Fig. 4 ). No procedure-related adverse events occurred, and histopathology confirmed the adenocarcinoma ( Fig. 5 ).

**Fig. 2:**
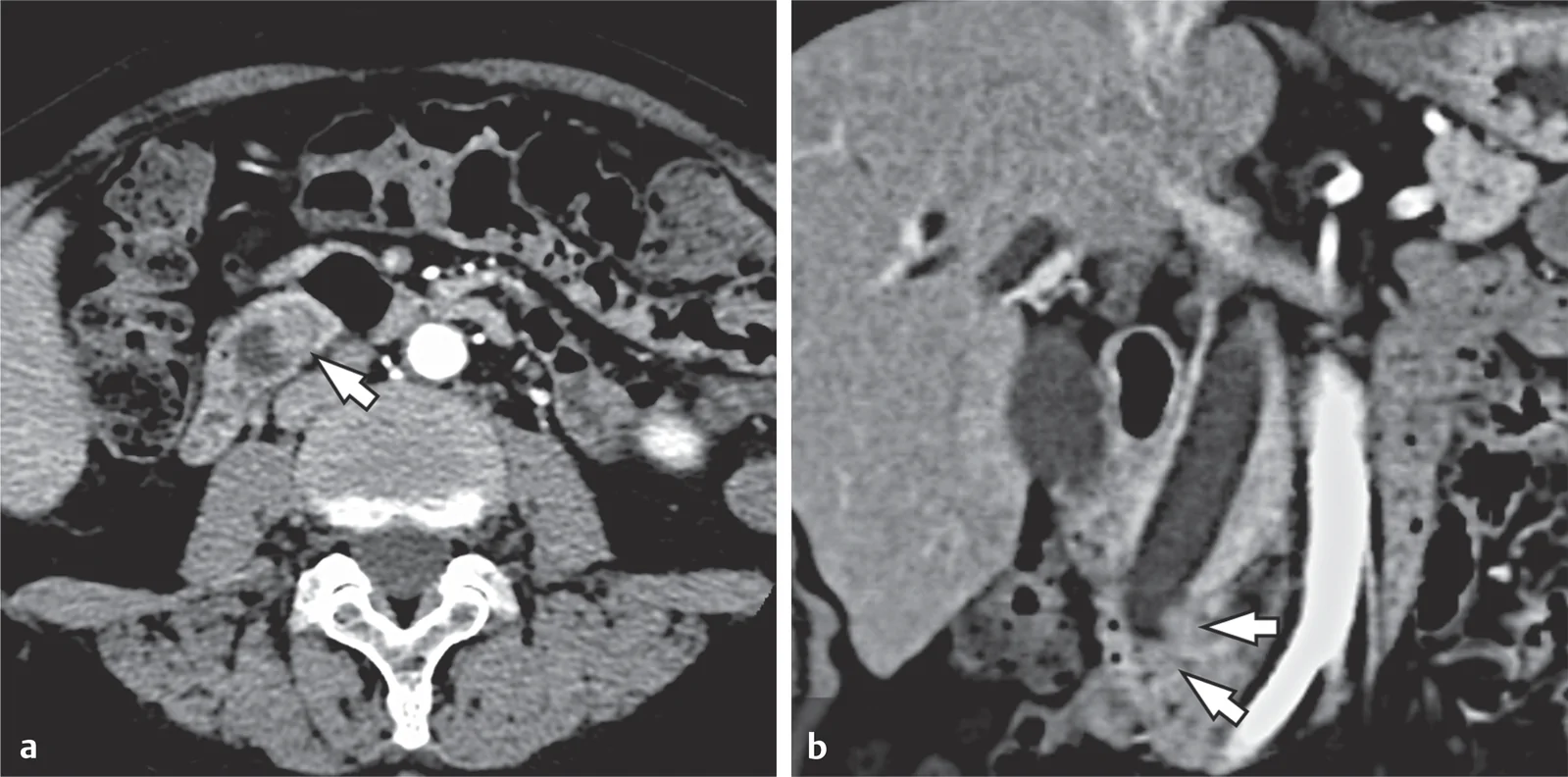
Contrast-enhanced computed tomography findings. ( **a** ) An axial image showing a tumor filling the distal bile duct and main pancreatic duct (white arrow). ( **b** ) A coronal image showing distal biliary obstruction caused by the tumor (white arrows), with diffuse bile duct dilatation.

**Fig. 3:**
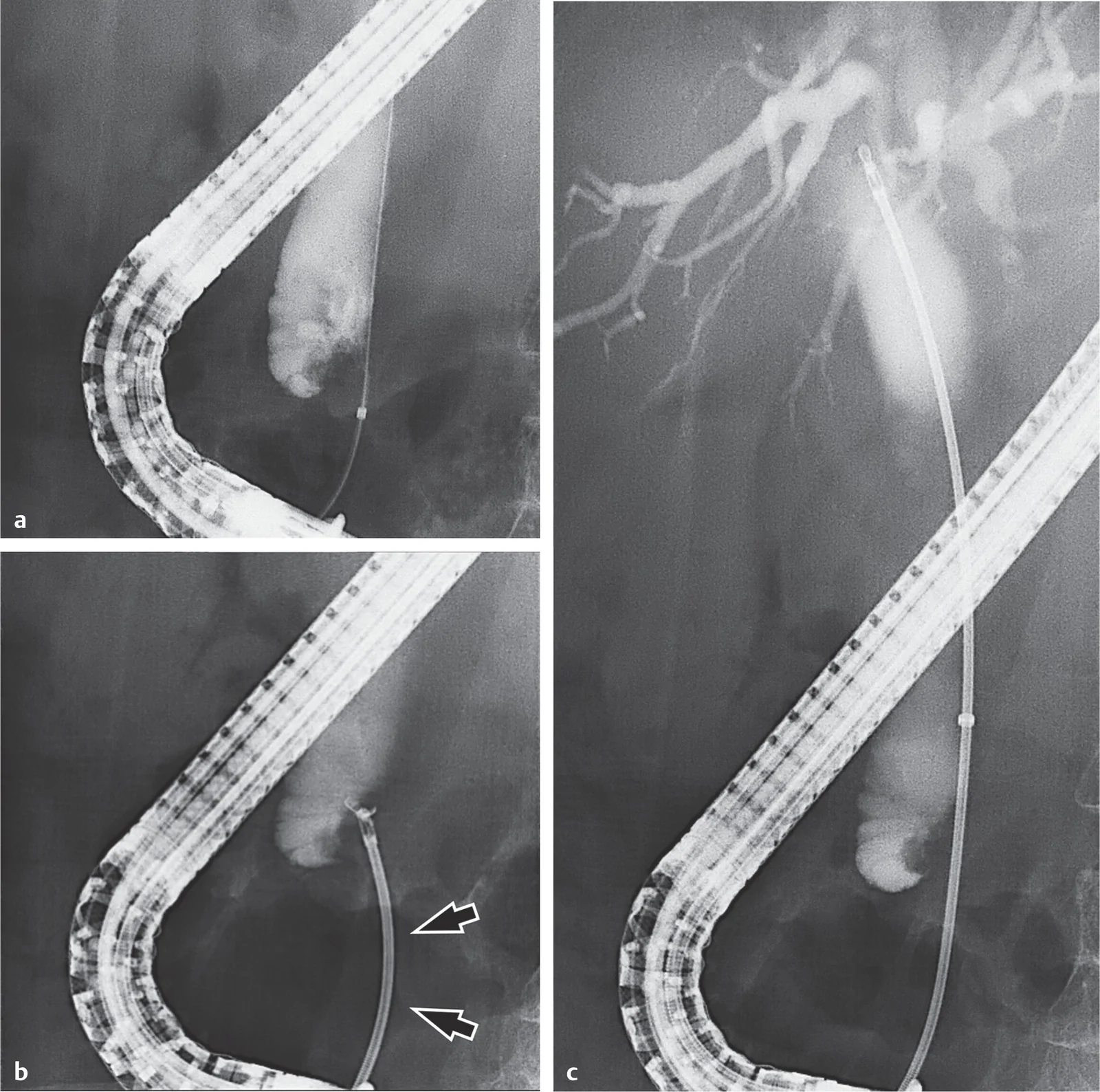
ERCP findings and the “flex-and-resheath” technique. ( **a** ) Cholangiography showing a filling defect extending from the ampulla to the distal bile duct. ( **b** ) Temporary sheath withdrawal allowed a curved forceps trajectory (black arrows) toward the distal biliary tumor. ( **c** ) Subsequent resheathing over the forceps maintained biliary access.

**Fig. 4:**
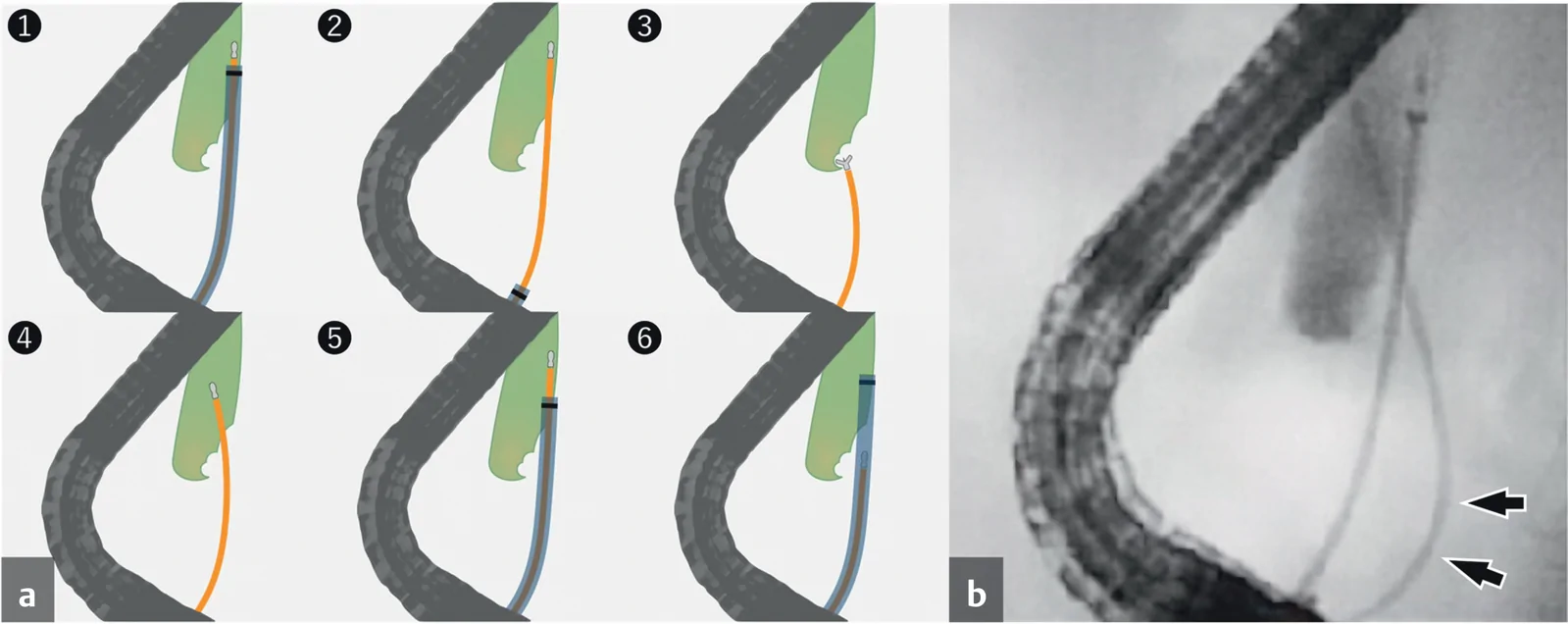
A schematic sequence of the “flex-and-resheath” technique and fluoroscopic overlay comparing forceps-shaft flexibility. ( **a** ) Standard-sized forceps are inserted into the bile duct through the outer sheath of EndoSheather (a1). With the forceps retained in the bile duct, the outer sheath is temporarily withdrawn into the endoscope (a2), restoring forceps-shaft flexibility and allowing a curved trajectory toward the target lesion (a3). After grasping the tissue, the forceps are gradually advanced (a4), with subsequent resheathing over them (a5). The forceps with the specimen are retrieved through the outer sheath, preserving biliary access for repeated biopsies (a6). ( **b** ) The overlay of fluoroscopic reference frames compares the sheathed and unsheathed configurations, showing restored forceps-shaft flexibility (black arrows).

**Fig. 5:**
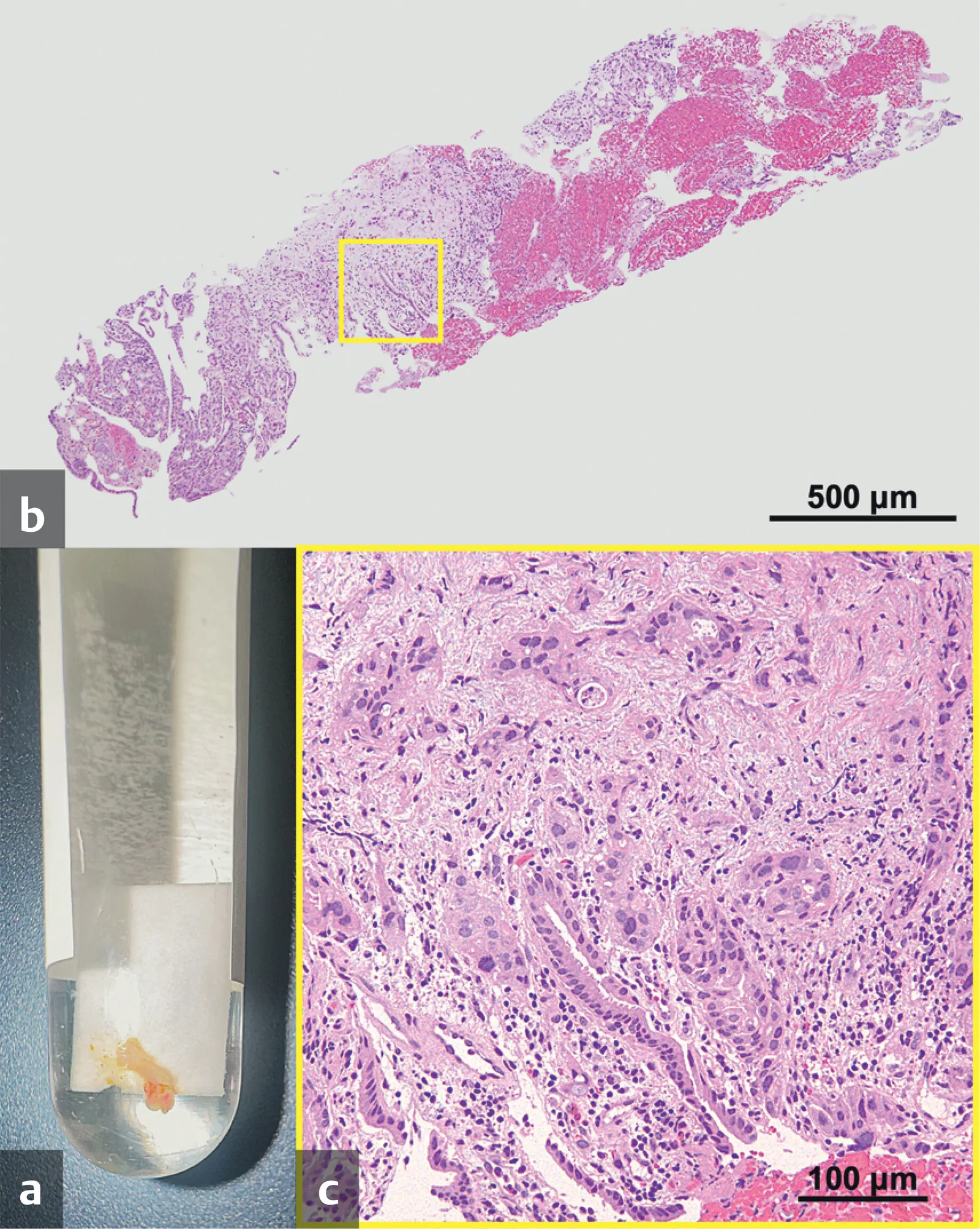
The biopsy specimen and histopathological findings. ( **a** ) The tissue obtained in one forceps bite using the “flex-and-resheath” technique was macroscopically adequate; the filter paper is approximately 10 mm wide. ( **b** ) A low-power overview of the entire hematoxylin and eosin-stained biopsy specimen. (c) A higher-power view showing irregular glands composed of atypical epithelial cells infiltrating the fibrous stroma, confirming the adenocarcinoma.

Conventional forceps insertion may be difficult when traversing biliary strictures, and repeated insertion may increase papillary stress. 1​ ​3​ The EndoSheather facilitates repeated biopsies through a stable biliary access route. 2​ ​3​ However, the sheathed configuration may restrict forceps flexibility in the distal bile duct, limiting tissue sampling from these lesions. 2​ The “flex-and-resheath” technique restores forceps flexibility in the unsheathed configuration while preserving stable biliary access provided by the sheath. This simple maneuver may broaden the applicability of sheath-assisted biopsy to anatomically challenging lesions.

Endoscopy_UCTN_Code_TTT_1AR_2AD

## Declaration of GenAI Use

The authors confirm that no generative artificial intelligence was used in the preparation of this manuscript.
